# Dexmedetomidine Efficacy in Quality of Surgical Field During Endoscopic Sinus Surgery

**Published:** 2019-09

**Authors:** Arman Parvizi, Soudabeh Haddadi, Ali Faghih Habibi, Shadman Nemati, Nikoo Akhtar, Hedieh Ramezani

**Affiliations:** 1 *Anesthesiology Research Center,* * Department of Anesthesiology* *, Alzahra Hospital ,Guilan University of Medical Sciences, Rasht, Iran.*; 2 *Otorhinolaryngology Research Center, Department of Otolaryngology and Head and Neck Surgery, School of Medicine, Guilan University of Medical Sciences, Rasht, Iran.*; 3 *Student of Medicine,* *Guilan University of Medical Sciences, Rasht, Iran.*

**Keywords:** Dexmedetomidine, FESS, Functional endoscopic sinus surgery, Hemodynamic stability, Intraoperative bleeding

## Abstract

**Introduction:**

Blood loss is a common concern during functional endoscopic sinus surgery (FESS). The present study aimed to evaluate the efficacy of dexmedetomidine (DEX) in intraoperative bleeding and surgical field in FESS.

**Materials and Methods:**

This double-blind randomized clinical trial was conducted on 72 patients within the age range of 16-60 years who underwent FESS. The subjects were randomly dividedinto two groups. The DEXgroup received 1 mic/kg DEX in 10 min at anesthesia induction followed by 0.4 to 0.8 mic/kg/hour during maintenance, while the control group received normal saline instead of DEX in bolus with the same volumemaintenance. Heart rate, systolic blood pressure, diastolic blood pressure (DBP),mean arterial pressure (MAP),and opioid requirement were evaluated in the 15^th^, 30^th^, 60^th^, and 90^th^min of the induction. The surgeon's assessment of the field during surgery and intraoperative bleeding was also recorded in this study.

**Results:**

The DEX group had lower bleeding scores (P=0.001) than the control group.Surgeon's satisfaction based on a Likert scale (P=0.001) was lower in the control group. The mean of DBP was lower in the DEX group in the 30^th^(P=0.001), 60^th^(P=0.001), and 90^th^(P=0.01) min of the induction. The MAP was lower in the DEX group in the 30^th^(P=0.015), 60^th^(P=0.052), and 90^th^(P=0.046) min of the induction. There were no postoperative adverse effects in the DEX group.

**Conclusion:**

It was observed that DEX improves the quality of the surgical field and hemodynamic stability. In addition, DEX might be safely and effectively used in surgeries in which deliberate hypotension is desirable.

## Introduction

Sinus surgery is one of the most prevalent surgeries on ear, nose, and throat (ENT), which is mainly carried out nowadays through endoscopy and leads to significant improvement in the clinical symptoms of patients with rhinosinusitis (1). It is necessary to maintain safe conditions for this surgery, and the major problem reported during functional endoscopic sinus surgery (FESS) under general anesthesia (GA) is impaired visibility due to excessive amounts of bleeding (2,3). This is particularly important for the successful surgery of the ethmoid and sphenoid sinuses because even minimum amount of bleeding might seriously impair the successful completion of the surgery and increase the operational risk and time (3,4), which is a major concern for both anesthesiologist and ENT specialist (5). 

Although excessive bleeding is rare, a desirable surgical field is often difficult to create. Even slight bleeding could cause abnormal endoscope vision and increased complications, including damage to the internal carotid artery, surgeon’s impaired vision, and as a result frequent use of suction. The increased manipulation and further bleeding would prolong the surgical operation and even make a surgical operation useless or abort the operation due to bleeding (6,7).

Anesthesiologists, in addition to causing analgesia and immobility in patients during the surgical operation, would prepare an appropriate condition to create a desirable surgical field for the better visibility of the surgeon; thereby, damaging arterial branches and other adjacent anatomical structures is reduced, which will lead to decrease surgical time (8). Intraoperative bleeding may be effectively reduced by decreasing systemic blood pressure. There are several important advantages for the anesthesia technique of controlled hypotension in FESS, such as lowering bleeding rate as the result of lowered blood transfusion rate, improved surgical field vision, and reduced duration of the operation (4). Several pharmaceutical methods can be used in order to achieve deliberated hypotension. Nitroglycerine (4,9) is one of the most widely used drugs for controlled hypotension; however, despite decreasing blood pressure, it practically leads to increased bleeding in the surgical area due to vasodilation. The use of high doses of inhaled anesthetics, sodium nitroprusside, vasodilators, and β-blockers is among other methods that can be utilized alone or sometimes combined with other techniques (4,10). It seems that the ideal drug for controlled hypotension should have certain characteristics, such as short onset time, fast removal without toxic metabolites, easy injection, disappearance of effects after withdrawal, and dose-dependent predictable effects (4). 

Dexmedetomidine (DEX) is an imidazole, active dextro-isomer compound of medetomidine, and most specific selective α^2^-adrenergic receptor agonist. This agonist has a tendency that is eight times higher than clonidine for α^2^-adrenergic receptor (3,4,11). The beneficial dose-dependent effects of DEX are sedation, analgesia, sympatholysis, and reduced anxiety without respiratory depression. The DEX has a dual-phase and dose-dependent response to blood pressure. Although its high dose leads to an increase in blood pressure caused by activating α^2^- and β-adrenergic receptors in the smooth muscles of the vessels, the dominant activity of α^2^-adrenergic receptor agonist with low concentration lowers blood pressure (3,11). 

In this regard, despite the usage of DEX in some studies during sinus endoscopy, tympanoplasty, and septoplasty, DEX is an expensive drug and isn't easily available but in the endoscopic sinus surgery, it's essential to use an agent for deliberated hypotension,. Therefore, given the numerous cases of sinus endoscopy and constant challenge for controlling intraoperative bleeding (4), the present study investigated the effect of DEX as a hypotensive factor to measure the bleeding rate and quality of surgical field among the patients undergoing FESS in a specialized center of ENT diseases in Rasht, Guilan Province, Iran. 

## Materials and Methods

The present study was a double-blind randomized controlled clinical trial approved by the Ethical Committee of Guilan University of Medical Sciences, Guilan, Iran (IR.GUMS.REC.1395.75). The clinical trial registration code was obtained from the registration center of clinical trials (201606211138N23), and written informed consent was acquired from all the participants. The present study was conducted on 72 patients within the age range of 16-60 years old who consecutively underwent FESS under GA by the same surgeon between July 2016 and April 2017. According to the guidelines of Academy of Otolaryngology-Head and Neck Surgery, the exclusion criteria were pregnancy, hemoglobin level lower than 10 mg/dL, abnormality of Prothrombin Time, Partial Thromboplastin Time or International Normalized Ratio, history of allergy to anesthetic or other drugs, coagulation defects or using anticoagulants, such as heparin 48 h before the surgery, acute and chronic renal failure, diabetes and cerebrovascular diseases, cerebrovascular insufficiency, deep vein thrombosis, peripheral venous disease, history of respiratory problems, and known systemic or psychiatric disorders. A computer-generated randomization file was used to assign the patients into two groups of DEX and control.

The participants, operation nurse, and ENT surgeon constituted the blind study group. The anesthesiologist performing the research was unaware of the constituents of the drug fitted with a syringe pump and allotment of the group; similarly, the residents keeping records of different parameters were also unaware of group assignment. Therefore, the blinding process was properly preserved. In the Dex group (n=36), the patients received the DEX loading dose of 1 μg/kg in 10 ml of diluted 0.9% saline solution during 10 min before anesthesia followed by the infusion of 0.4-0.8 μg/kg. The subjects in the control group (n=36) received normal saline infusion as a bolus, maintenance dose, and then the drugs required for the induction of anesthesia without DEX. In both groups, the infusion rate was adjusted to maintain the mean systolic blood pressure (SBP) of 85-90 mmHg. The anesthesia protocol was the same for both groups as follows: midazolam (0.20 mg/kg), fentanyl (2 μg / kg), and lidocaine (1.5 mg/kg) were prescribed after the control of vital signs and establishment of monitoring. The anesthesia induction was performed using propofol (2.5 mg/kg) and atracurium (0.5 mg/kg). 

After the intubation, anesthesia continued with the infusion of propofol (100 μg/kg/min) and remifentanil (0.1 μg/kg/min). Controlled ventilation was performed by nitrous oxide at a concentration of 50% and O2. After the end of the surgery, the muscle reverse relaxation was carried out by neostigmine 0.04 mg/kg and atropine 0.02 mg/kg. Before the operation, 3 ml/kg of the isotonic fluid was infused. Moreover, during the operation, IV was given to the patients depending on the weight of the patient and bleeding. Both drugs in the present study were diluted in an equal amount of normal saline in syringe pumps for blinding purposes.

A concise questionnaire was administered that collected demographic data. During the procedure, several parameters were measured noninvasively and continuously and then recorded as follows:

The SBP, diastolic blood pressure (DBP), heart rate (HR), and mean arterial pressure (MAP) in the intervals of 15, 30, 60, and 90 min were measured, as well as the satisfaction level of the surgeon based on a Likert scale (5: excellent, 4: Good, 3: Satisfactory, 2: Poor, 1: Very poor) (12). Surgical satisfaction was evaluated by one surgeon and recorded according to a 5-point Likert scale (5: excellent, 4: Good, 3: Satisfactory, 2: Poor, 1: Very poor)

Intraoperative bleeding was estimated by the surgeon via rating the amount of bleeding based on Boezzart's scale for the evaluation of operative field visibility during the surgery demonstrating 0: no bleeding, 1: slight bleeding, in which blood evacuation is not necessary; 2: slight bleeding, in which some blood should be evacuated; 3: light bleeding, in which blood should be frequently evacuated as operative field is visible only briefly after the evacuation; 4: average bleeding, in which blood should be often evacuated as the operative field is visible only immediately after the evacuation; and 5: vigorous bleeding, in which constant blood evacuation is needed as bleeding often exceeds the evacuation resulting in rendering the surgery nearly impossible (2,3). 

The operation starting and ending times were written down, and postoperative adverse effects, such as nausea, vomiting, bradycardia, tachycardia, and hypotension, were recorded. The Mann-Whitney U test was used in order to compare the amount of bleeding and level of satisfaction. The independent t-test was employed in both groups (a nonparametric counterpart test was used in case of nonnormal distribution of the above-mentioned quantitative variables) to compare the quantitative variables during the surgery, including SBP, DBP, and HR.P-value less than 0.05 was considered statistically significant. All the statistical procedures were performed in SPSS software (version. 22.0).

## Results

This study was performed on a total of 72 patients who were candidates for endoscopic sinus surgery.The subjects were divided into two groups of DEX and placebo with the mean age of 38.39±11.84 and 42.94±13.46 years, respectively.There was no statistically significant difference in terms of age and sex between the two groups (Table.1).

**Table 1:** Comparison of demographic data and operative times between two studied groups

	**Dexmedetomidine group(n=36)**	**Control group(n=36)**	**P-value**
Age* (years)	38.39±11.84	42.94±13.46	0.132
Gender (male/female)	23/13	21/15	0.629
Operative time* (min)	86.67±17.03	85.14±15.19	0.636

* Mean±standard deviation

There was no statistically significant difference between the two groups regarding the duration of the surgery. The mean value of surgical duration was 85.9±16 min with the minimum and maximum of60 and 135 min, respectively. In case of bleeding, a significant difference was observed between the two groups (P<0.001). In the control group, bleeding rates in grades 3 and 4 were significantly higher than those in the DEX group (77.7% and 33.3%) (Table.2). 

**Table 2:** Bleeding cores based on Boezzart's scale

	**Dexmedetomidine group**		**Control group**		**P-value**
	n	%	n	%	0.001
0	0	0.0	0	0.0	
1	10	27.8	1	2.8	
2	14	38.9	7	19.4	
3	9	25.0	21	58.3	
4	3	8.3	7	19.4	
5	0	0.0	0	0.0	

In the comparison of the bleeding state among men in both groups, the statistical difference was significant (P<0.001), while it was not significant among women (P=0.25). In the comparison of the bleeding state in the age group below 40 years, the statistical difference was marginal (P=0.05), and in the age group over 40 years, the difference was statistically significant (P=0.001). Generally, there was a significant disagreement between the two groups in terms of surgeon's satisfaction that was higher in the DEX group than that in the control one (P=0.001) (Table.3). In the comparison of surgeon's satisfaction among men in both groups, the statistical difference was statistically significant (P<0.001), while it was not significant among women (P=0.061). A statistically significant difference was observed in the age groups below and over 40 years (P>0.05).

**Table 3:** Surgeon’s satisfaction based on Likert scale

	**Dexmedetomidine group**		**Control group**		**P-value**
	n	%	n	%	0.001
Very bad	0	0.0	0	0.0	
Bad	0	0.0	2	5.6	
Moderate	3	8.3%	15	41.7	
Good	16	44.4	17	47.2	
Very good	17	47.2	2	5.6	

**Fig 1:**
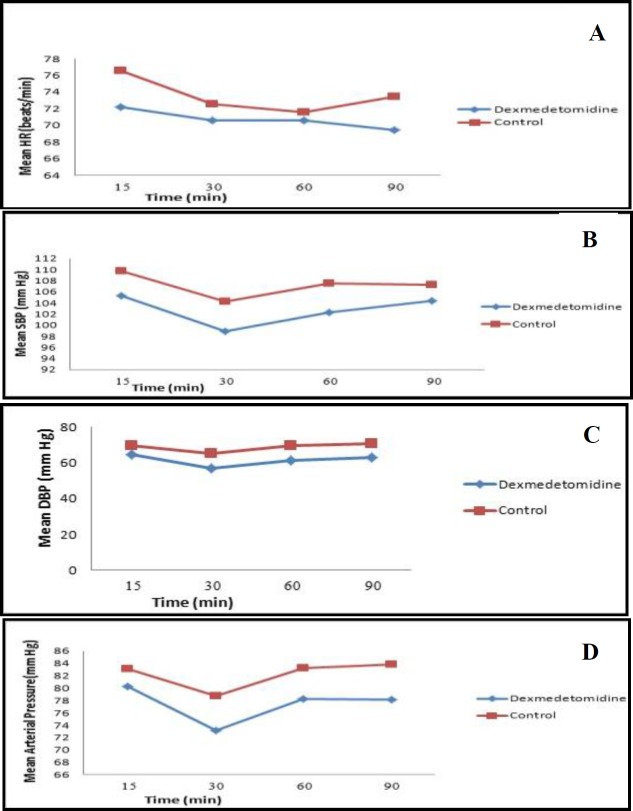
Comparing trend of changes in mean of heart rate (HR), (a) mean of systolic blood pressure (SBP), (b) mean of diastolic blood pressure (DBP), (c) mean of arterial blood pressure, (d) between dexmedetomidine and control groups in induction and different times after initiation of surgery

In Figure 1, according to the results of the within-group comparison in the DEX group, HR changed from 15 to 90 min with an almost constant trend; however, it was not statistically significant. In this regard, the change was significant in the placebo group (P=0.02). In general, the two studied groups were reported with no significant difference in HR at the measurement times (P=0.263). Furthermore, no significant difference was observed in HR between the two groups (P<0.05) divided by measurement times. The trend of HR changes in the two studied groups was the same during the admission periods with no significant difference (P=0.207).In the DEX group, there was a statistically significant difference in SBP changes from 15 to 90 min (P=0.044); however, these changes were not significant in the placebo group (P=0.222). Generally, the two studied groups had no significant difference in SBP at the measurement times (P=0.063), and no significant difference was observed in SBP between the two groups (p<0.05) divided by various measurement times. The trend of SBP changes in the two studied groups was the same during the admission periods, and there was no significant difference in this regard (P=0.835).

In the within-group comparison of the DEX (P<0.001) and control (P=0.052) groups, there was a statistically significant difference in DBP changes from 15 to 90 min. In the DEX group, DBP was lower than that of the control group at the measurement times; however, this difference was only significant at 30 (P=0.001), 60 (P=0.001), and 90 (P=0.010) min. There was a statistically significant difference between the two groups in terms of DBP (P<0.001). The trend of DBP changes in the two studied groups was the same during the admission periods, and there was no significant difference in this regard (P=0.586). 

In the within-group comparison of the DEX group, there was a statistically significant difference in MAP changes from 15 to 90 min (P=0.007); however, these changes were not significant in the placebo group (P=0.096). The MAP in the DEX group was lower than that of the control group during the measurement times. Nonetheless, this difference was only significant at 30 (P=0.015), 60(P=0.052), and 90 (P=0.046) min. In general, there was a significant difference between the two groups in terms of MAP (P=0.011). The trend of MAP changes in the studied group was the same during the admission periods with no significant difference (P=0.700).

## Discussion

The FESS is performed to treat chronic rhinosinusitis that is resistant to medical therapy. Since nasal and sinus mucus has many vessels, one of the most common concerns in endoscopy surgery is bleeding control (2). The DEX has a dual-phase and dose-dependent response to blood pressure. In addition, the dominant activity of α^2^-adrenergic receptor agonist with a low concentration is considered to reduce blood pressure (3,11). 

Several studies have reported the effectiveness of DEX in providing better surgery and lowering bleeding amount during controlled hypotension in tympanoplasty, septoplasty, and rhinoplasty (9,13). In the present study, the mean score of bleeding in the DEX group was significantly lower than that in the placebo group, and the level of surgeon’s satisfaction in the DEX group was significantly higher than that in the control group. Goksu et al. and Guven et al. reported better hemodynamic status (3,14), more analgesia, and better surgical field quality with fewer adverse effects in the DEX group than those of the control group during FESS. 

According to the results of a study carried out by Praveen et al. (15), there was a statistically significant reduction in HR in the DEX group, compared to that in the nitroglycerin group, and the patients in the DEX group required less intraoperative opioid. In another study conducted by Das et al., DEX was introduced as a more effective drug for controlled hypotension and anesthesia, compared to esmolol, consequently resulting in less nose bleeding and higher surgeon’s satisfaction score, which could lead to lower need for nitroglycerin and fentanyl as additional hypotension drugs. 

Overall, in the present study, the trend of changes in HR (P=0.207), SBP (P=0.835), DBP (P=0.586), and MAP (P=0.700) was the same during admission periods with no significant difference. The levels of DBP and MAP in the DEX group were significantly lower than those of the control group at the minutes of 30, 60, and 90. The patients in this study who used DEX had a significant decrease in DBP and MAP after 30 min of using the loading dose. In a study conducted by Shams et al., which was performed to compare the efficacy of DEX as a blood pressure decreasing factor and esmolol in FESS, there was a significant decrease in HR and MAP after 10 min of using the loading dose in the DEX group (9).

The main concern of GA is to reduce intraoperative bleeding by lowering blood pressure during the operation (13). In optimal anesthetic techniques, it seems that relative bradycardia is associated with blood pressure. The results of numerous studies have shown that the use of DEX is significantly associated with the reduced use of inhalation drugs, fentanyl, and analgesics in a dose-dependent manner (14). In a study carried out by Shams et al. (9), the propofol induction dose was significantly lower in the DEX group than that in the esmolol group. This finding is consistent with the results of a study by Peden et al. (16), which proposed DEX as a drug that can reduce the dose of propofol required to decrease consciousness. 

A study was conducted by Eghbal et al. in Arak (1), Iran (2017) on 100 patientsto compare the effect of labetalol and DEX on lowering intraoperative bleeding and FSE field conditions. In the aforementioned study, the bleeding score, extubation time, and recovery time in the labetalol group were reported to be lower than those in the DEX group. Moreover, the patients in the labetalol group had a higher recovery rate and lower amount of bleeding. In the aforementioned study, there was no statistically significant difference in the mean time between the two groups that is similar to the findings of the present study. 

The mean HR in the DEX group was significantly higher at all postinduction times (P<0.001). Moreover, a significant decreasing trend in mean HR was observed in the labetalol group, while HR was increasing in the DEX group. In the present study, in the within-group comparison of the DEX group, the HR changes were almost constant from the minutes of 15 to 90 and were not statistically significant; however, these changes were significant in the control group (P=0.02), and the mean HR in the DEX group was lower than that in the econtrol group at all measurement times. At various measurement times, no significant difference was observed in HR between the two studied groups (P <0.05). Furthermore, the trend of HR changes in the two studied groups was the same during admission periods with no significant difference (P=0.207).

In the present study, although the mean HR in the DEX group was significantly higher at all the postinduction times, the post-induction lowering blood pressure was observed in both groups. In the aforementioned study, labetalol was proposed as a drug that could reduce bleeding during FESS and improve the surgeon’s visibility with higher advantages over DEX in terms of lowering bleeding, extubation time, and recovery time. In the aforementioned study, no adverse effects, such as vomiting/nausea and chills, were reported in the DEX group, while 11% of the patients in the labetalol group experienced such adverse effects. On the other hand, no adverse effect was reported in the two groups in the present study. 

The effects of DEX have been evaluated, compared to other anesthetic drugs, such as esmolol, bisoprolol, magnesium sulfate, and isoflurane-alfentanil, in reducing blood loss, as well as improving surgery conditions, during FESS in some recent studies (1, 17, 18). In a study carried out by Bajwa et al. (18), which was performed on 150 patients (50 subjects per group) in 2016 to compare the effects of DEX, esmolol, and nitroglycerin on hypotension control among the patients undergoing FESS, it was reported that DEX and esmolol could be more effective in providing better hemodynamic stability and increasing the surgical field vision than nitroglycerin during FESS. Furthermore, postoperative sedation and reduced analgesic requirements were reported for DEX, compared to the other two drugs.

 Hadavi et al. (19) reported no statistically significant difference in the amount of bleeding during FESS between labetalol and nitroglycerin groups. The results of some studies, such as the one conducted by Kim et al. (20), revealed that in terms of surgeon’s satisfaction and bleeding in FESS, DEX was not different from remifentanil; however, the extubation time in the DEX group was shorter. A study by Lee et al. was performed on 66 patients to compare the efficacy of two drugs, namely DEX and remifentanil hydrochloride, in intraoperative surgical field status and sinus endoscopic surgery recovery. No significant difference was observed in total lost blood, surgical field conditions, hemodynamic parameters, and extubation time between the two groups (P>0.05).

Although both above-mentioned drugs were capable of lowering blood pressure and providing good field vision during the sinus endoscopic surgery, the postoperative immediate recovery of remifentanil was reported to be faster (21). In the aforementioned study, similar to the present one, propofol was used to induce anesthesia, and DEX was infused with the loading dose of 1 μg/kg before inducing anesthesia and a remifentanil dose of 0.4-0.8 mg/kg for maintenance. In the aforementioned study, in the remifentanil group, the dose of 1 μg/kg was used for induction similar to the present study; however, the dose of 0.2-0.4 μg/kg was infused for maintenance. In addition, it can be said that the faster recovery of patients in the postanesthesia care unit observed in the aforementioned study was due to the greater infusion dose of remifentanil, compared to that in the present study. Bajwa et al. reported that better recovery duration can be achieved using DEX (18). In a study carried out by Shams et al., there was no significant difference between DEX and esmolol groups in terms of MAP and HR. As far as surgery field quality was concerned in the MAP range (55-65 mmHg) were below or equal to 2, no statistically significant difference was observed during the course of lowering blood pressure; however, the average intraoperative intake of fentanyl was considerably lower in the DEX group. 

In the above-mentioned study, the sedation score was significantly lower at 15^th^ and 30^th^ min after the surgery in the esmolol group than that in the DEX. Moreover, the first demand for analgesics in the DEX group was considerably longer, and the recovery was significantly faster. In general, although both drugs were desirable to control blood pressure reduction and provide surgical field during FESS, DEX was proposed as an alternative to esmolol with the advantages of analgesic, sedative, and anesthetic-sparing effects (9).

The results of another study showed that DEX led to a significant reduction in the amount of bleeding and level of HR in FESS, compared to magnesium sulfate (23). In a study conducted by Guven et al., the mean bleeding score in the DEX group was reported significantly lower than the that in the control group (P=0.019) that is consistent with the finding of the present study. The HR in the DEX group, similar to the present study, was reported to be lower than that in the control group during all the recorded surgical periods (1^st^, 5^th^, 10^th^, 20^th^, 30^th^, 45^th^, and 60^th^ min); however, the difference was significant only at the anesthesia induction time (P=0.019), 1 (P=0.09) and 20 (P=0.039).

Nonetheless, in the present study, no significant difference was observed in HR between the two groups (P>0.05) at different measurement times. Furthermore, there were no adverse effects caused by drug administration. Guven et al.reported the postoperative side effects, such as nausea, vomiting, tachycardia, and hypotension (mean blood pressure lower than 50 mm Hg), in the DEX group had a lower frequency than those in the control group; however, the difference was statistically significant (P<0.001). Indeed, these patients required no intervention (14). There were some limitations in the present study one of the most important of which was the high price of DEX resulting in the restriction of drug usage. In short, it was observed that DEX improved the surgical field with a high score of surgeon’s satisfaction, reduced HR and mean BP at a balanced level, and decreased the postoperative side effects, such as nausea and vomiting.

## Conclusion

The DEX might be safe and effective in surgical operations, such as FESS, in which the controlled hypotension is desirable.Generally, DEX would provide excellent surgery with a high score of surgeons’ satisfaction and little need for additional hypotensives(i.e. nitroglycerin and fentanyl) almost without severe hemodynamic changes and adverse effects. It is recommended to perform further studies with larger sample size andcomparison between DEX,esmolol, and Labetolol in patients who are candidates for endoscopic sinus surgery. However, these drugs arenot available now.
